# Synergistic action of dual IGF1/R and MEK inhibition sensitizes childhood acute lymphoblastic leukemia (ALL) cells to cytotoxic agents and involves downregulation of STAT6 and PDAP1

**DOI:** 10.1016/j.exphem.2018.04.002

**Published:** 2018-07

**Authors:** Victoria J. Weston, Wenbin Wei, Tatjana Stankovic, Pamela Kearns

**Affiliations:** a Institute of Cancer and Genomic Sciences, University of Birmingham, Edgbaston, Birmingham B15 2TT, UK; b Sheffield Institute of Translational Neuroscience, University of Sheffield, Sheffield S10 2HQ, UK

## Abstract

Heterogeneous upregulation of multiple prosurvival pathways underlies resistance to damage-induced apoptosis in acute lymphoblastic leukemia (ALL) cells despite normal p53 responses. Here, we show that the dual combination of insulin-like growth factor 1 (IGF1)/IGF1 receptor (IGF1/R) and mitogen-activated protein kinase/extracellular signal-regulated kinase (ERK) kinase (MEK) inhibition using AG1024 + U0126 can sensitize apoptosis-resistant ALL cells to ionizing radiation-induced DNA damage irrespective of effect of single pathway inhibition in vitro. This AG1024 + U0126 combination also significantly potentiates the ability of the core chemotherapy compounds vincristine, dexamethasone, and daunorubicin to kill ALL cells in vitro*.* Evidence of the synergistic action of AG1024 + U0126 in samples with variable basal levels of phosphorylated IGF1/Rβ and ERK1/2 suggested additional targets of this drug combination. Consistent with this, gene expression profiling identified 32 “synergy genes” differentially targeted by IGF1/R + MEK inhibition and, among these, *Signal transducer and activator of transcription 6 (STAT6)* and *platelet-derived growth factor-associated protein 1 (PDAP1)* were the most differentially downregulated cluster. Pearson correlation analysesrevealed that *STAT6* and *PDAP1* display significant expression codependency and a common expression pattern linked with other key “synergy” genes, supporting their predicted role in an STAT6–ERK–nuclear factor kappa beta (NF-κB) network. Knockdown studies revealed that loss of STAT6, but not PDAP1, impinges on the cell cycle, causing reduced numbers of viable cells. In combination with daunorubicin, STAT6 loss has an additive effect on cell killing, whereas PDAP1 loss is synergistic, indicating an important role of PDAP1 in the cellular response to this anthracycline. Inhibition of STAT6 or PDAP1 may therefore represent a potential novel therapeutic strategy for resistant ALL by enhancing sensitivity to chemotherapy.

## Highlights

• Dual insulin-like growth factor 1 receptor (IGF1/R) + mitogen-activated protein kinase/extracellular signal-regulated kinase (ERK) kinase (MEK) inhibition synergistically sensitize apoptosis-resistant acute lymphoblastic leukemia (ALL) cells.
• Dual IGF1/R + MEK inhibition potentiates chemotherapy-induced killing of ALL cells.
• Signal transducer and activator of transcription 6 (STAT6) and platelet-derived growth factor-associated protein 1 (PDAP1) are downregulated differentially by this synergistic action.
• STAT6 and PDAP1 are predicted to act in a putative STAT6–ERK–nuclear factor kappa beta (NF-κB) survival network.
• Targeting this network may be useful for sensitizing ALL to chemotherapy agents.

Although cure rates for childhood acute lymphoblastic leukemia (ALL) have increased dramatically over recent years through the integration of risk stratification into treatment protocols 1, 2, 3, failure of remission-inducing therapy is associated with only a 30% 10-year survival rate [4] and relapsed ALL remains the most common cause of cancer-related death in children 5, 6, 7. There is a need for new therapeutic approaches with minimal toxicities for higher-risk leukemias.

Philadelphia-positive (Ph+) ALL is currently the only ALL subset for which treatment includes molecularly targeted treatment and the combination of imatinib with chemotherapy has significantly improved survival rates in this subgroup [8]. Recent advances in the molecular characterization of childhood ALL has identified several new, risk-associated genotypes implicating specific prosurvival signaling pathways 8, 9, 10, 11, 12, 13. Although these pathways potentially encompass multiple molecular targets, the evident heterogeneity could complicate a personalized treatment approach for ALL patients. For example, hyperactivation of the receptor tyrosine kinase and RAS signaling pathways is a prominent feature of hypodiploid ALL and these cells demonstrate sensitivity to phosphoinositide 3-kinase (PI3K), but not mitogen-activated protein kinase (MAPK)/extracellular signal-regulated kinase (ERK) kinase (MEK) inhibition in vitro [14]. In contrast, high-hyperdiploid ALL with activating *RAS* mutations is responsive to MEK inhibition in vitro [15]. Preclinical studies on adverse-risk Ph-like ALL, which frequently harbors *Janus kinase (JAK)* and *cytokine receptor-like factor 2 (CRLF2)* mutations, indicate sensitivity to JAK inhibition and mammalian target of rapamycin (mTOR)/PI3K inhibitors 10, 16, 17, whereas *platelet-derived growth factor beta (PDGFB)-* and *ABL*-rearranged Ph-like disease instead appear to display responsiveness to the tyrosine kinase inhibitors imatinib and dasatinib 8, 18, 19. Consistent with these findings, our previous work also showed that childhood ALL displaying both poor clinical outcome and impaired apoptotic responses to DNA damage in vitro exhibit heterogeneous upregulation of multiple prosurvival pathways, which notably involves the PI3K, insulin-like growth factor 1 (IGF1) and MAPK pathways 20, 21. Hyperactivation of these pathways, despite apparently normal p53 activation, causes sustained nuclear factor kappa beta (NF-κB) activity and impaired apoptosis after DNA damage [20]. Treatment with single prosurvival pathway inhibitors could only sensitize ALL cells to DNA damage in vitro in a patient-specific manner 20, 21.

Therefore, although it is clear that a significant proportion of ALL patients are likely to benefit from a molecularly targeted treatment added onto an existing chemotherapy regimen, the heterogeneous deregulation of signaling pathways paired with possible activation of compensatory pathways may hamper sensitization of ALL by single-pathway inhibition. We hypothesized that targeting dual pathways might be more effective against a broader spectrum of samples and indicate a more applicable therapeutic approach for patients with ALL showing impaired clinical responses. Here, we show that the specific combination of IGF1/IGF1 receptor (IGF1/R) and MEK inhibition can synergistically sensitize primary ALLs to a range of cytotoxic agents. We show that the mechanism of this drug combination involves downregulation of *signal transducer and activator of transcription 6 (STAT6)* and *PDGF-associated protein 1 (PDAP1)*, which appear to function in a predictedSTAT6–ERK–NF-κB regulatory network that may be implicated in apoptosis resistance in childhood ALL.

## Methods

### Patient ALL samples

Patient bone marrow (BM) samples were collected from Birmingham Children's Hospital with ethical approval and written consent (CCLG 08/H0405/22 and 08/H1208/4). Leukemic BM mononuclear cells were separated by density centrifugation and frozen in a viable state before use. For clinical data, see Supplementary Table E1 (online only, available at www.exphem.org).

**Supplementary Table E1:** Clinical features of paediatric ALL samples.

ALL	Subtype	Age (y)	Cytogenetics	WCC (50x10^9^/L)	MRD risk d28
S025117	cALL	3.1	ETV6-RUNX1	108	HR
S026767	T	7.08	n/k	121.4	nk
S027836	B	2.7	No results	13.5	LR
S029946	B	11	IgH@	10.1	HR
S029947	B	2	High hyperdiploid	9.3	HR
S032957	B	5.11	High hyperdiploid	10.2	HR
S038556	B	9.9	High hyperdiploid	33.8	LR
ALL-75	T	7	2xp16del	193	nk
ALL-102	cALL	7.02	Hyperdiploid (52)	62	HR
ALL-106	cALL	15	Near Haploid (28)	117	HR
ALL-111	cALL	10.09	Gain of AML1	2	HR
ALL-115	T	4.1	Myb^dup^	nk	LR
ALL-141	cALL	14	Gain of ETV6	11	HR
ALL-150	B	8	ETV6-RUNX1	nk	LR
ALL-200	B	4.06	nk	142	nk
ALL-201R	BCP-ALL	17.5	IGH@	na	na
ALL-202	cALL	6.08	46,XX Subclone of IGH@ gain	3	LR
ALL-203	cALL	2.10	CRLF2 rearranged	17.6	HR
ALL-211	T	7.05	TCRD-LM02	405	LR
ALL-212	T	5.07	46, XY	140	LR
ALL-213	T	8	SIL-TAL1	493	HR

Nk, not known; na, not applicable, HR, High risk; LR, Low risk.

### Annexin V/propidium iodide (PI) apoptosis analysis

Cells were treated with 5 Gy ionizing radiation (IR) and incubated with U0126 (Promega, WI, USA), AG1024, or LY294002 (Calbiochem, Darmstadt, Germany) at 37°C for 24hours, as described previously [21]. Apoptosis was assayed using an Annexin V Apoptosis Kit (BD Pharmingen, San Diego, CA) and analyzed using a Coulter Epics XL-MCL flow cytometer (Beckman Coulter, Fullerton, CA). Cells were considered to be apoptotic if they stained positive for both Annexin V and PI (Ann+/PI+). The proportion of IR-induced apoptotic cells was determined by subtracting the proportion of apoptotic cells detected in the absence of IR.

### Drug preparation and cytotoxicity assays

U0126, AG1024, LY294002, vincristine, and daunorubicin were dissolved in dimethylsulfoxide (DMSO) and dexamethasone in 100% ethanol at stock concentrations of 10mmol/L. Cells were incubated with U0126, AG1024, vincristine, daunorubicin, or dexamethasone (Sigma-Aldrich, St. Louis, MO, USA) at the indicated doses at 37°C for 72hours and subsequently reacted with Cell TitreGlo reagent according to the manufacturer's instructions (Promega). Luminescence was quantified using a Victor Wallac plate reader.

### Western blotting

Weston blotting was performed as described previously [21]. Antibodies included IGF1Rβ (#3027), phospho-IGF1Rβ (Tyr1131)/insulin receptor β (Tyr1146) (#3021), ERK1/2 (137F5), phospho-ERK1/2 (Thr202/Tyr204) (D13.14.4E), PDAP1 (#4300), procaspase 7 (D2Q3L), cleaved caspase 3 (5A1E) (Cell Signaling Technology, Danvers, MA, USA), STAT6 (ab44718) (Abcam, Cambridge, MA, USA), and PARP1 (Santa Cruz Biotechnology, Santa Cruz, CA, USA). Mouse monoclonal antibody against β-actin (AC-74) (Sigma-Aldrich) served as a loading control.

### Microarray analysis

Cells were untreated, treated with 5 Gy IR, 30 µmol/L AG1024 + 5 Gy IR, 20 µmol/L U0126 + 5 Gy IR, or 30 µmol/L AG1024 + 20 µmol/L U0126 + 5 Gy IR for 6hours in vitro before RNA extraction using a combined TRIzol/chloroform(Invitrogen)/RNeasy column purification (Qiagen) method as described previously [21]. After first- and second-strand synthesis and in vitro transcription, samples were hybridized to HuGene1.0 ST v1 gene chips (Affymetrix, Santa Clara, CA, USA). Probe-level quantile normalization [22] and robust multiarray analysis [23] on the raw .CEL files was performed using AltAnalyze [24]. Differentially expressed genes were identified using limma with a fold change >1.5 and *p* < 0.01 [25].

### siRNA silencing of STAT6 and PDAP1

Small interfering RNA (siRNA) silencing was performed in HeLa cells using SiGenome SMARTpools targeting human *STAT6* (6778) and human *PDAP1* (11333) (Thermo Scientific, Waltham, MA, USA). Two daily sequential siRNA treatments were performed using DharmaFECT transfection (Thermo Scientific) according to the manufacturer's instructions and nontargeting pool #2 (scrambled siRNA) served as a control. Data from three separate knockdown experiments are shown.

### Cell cycle analysis

Treated cells were fixed in 100% ice-cold ethanol before staining with PI (Sigma-Aldrich) and cell cycle profiles assessed using a Coulter Epics XL-MCL flow cytometer (Beckman Coulter).

### Statistical and network analysis

Combination indices (CIs) were determined using dose– response curves and Calcusyn software and potentiation effects (PEs) using paired Student *t* tests of data normalized to untreated cells and with a single-agent effect subtracted. Standard deviations are shown. Pearson correlation coefficients were determined by comparing normalized LOG2 expression values as described previously [26]. Network analysis was performed by seeding synergy genes using Ingenuity Systems IPA software (Qiagen) according to the manufacturer's instructions.

## Results

### Combined IGF1/R and MEK inhibition using AG1024 + U0126 sensitizes primary ALL cells to DNA damage-induced apoptosis

**Figure1:**
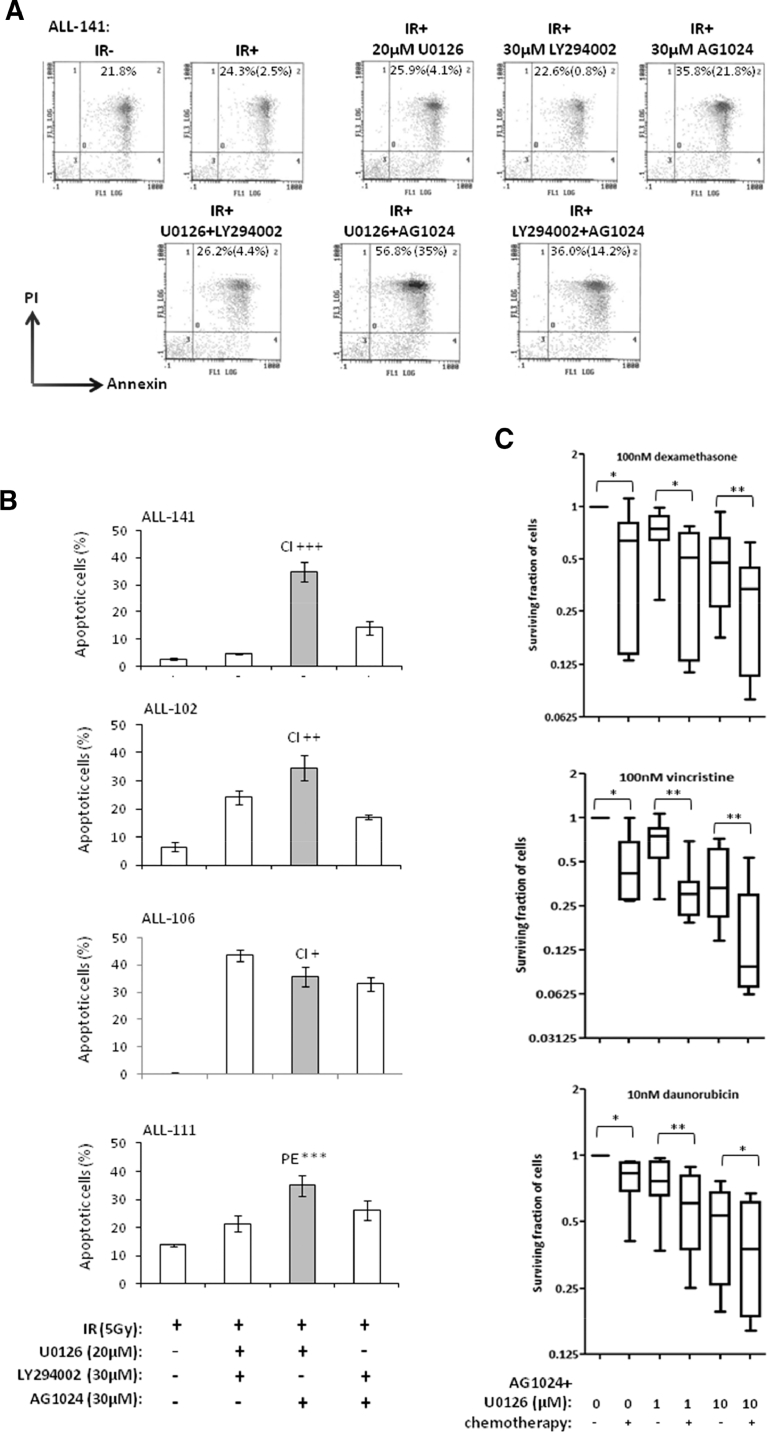
The combined inhibition of IGF1/R + MEK pathways by AG1024 + U0126 sensitizes ALL cells to cytotoxic agents. **(A)** Representative scatter plots of Annexin/PI staining in ALL-141 are shown. The average total percentage of apoptotic cells (Ann+/PI+, quadrant 2) induced by each treatment is shown, as well as the average IR-induced percentage of apoptosis (shown in parentheses), which were determined by subtracting the background apoptosis from the IR plots. **(B)** The combination of AG1024 + U0126 (shaded) consistently sensitizes ALL cells to IR-induced apoptosis regardless of the effect of AG1024 and U0126 as single agents in four cases (ALL-111, ALL-102, ALL-106, and ALL-141) after 24hours of treatment, as determined by Annexin V/PI staining. (The maximum concentration of DMSO reached in this experiment was 0.6% v/v DMSO.) CIs could be determined from individual drug dose curves (Supplementary Figure E3, online only, available at www.exphem.org) for ALL-102 (++, moderate synergism), ALL-106 (+, nearly additive), and ALL-141 (+++, synergism). For ALL-111, the PE of the combination of AG1024 + U0126 was determined using unpaired *t* test analysis as described (***, strong synergism) (Supplementary Table E2, online only, available at www.exphem.org). **(C)** AG1024 and U0126 together sensitize ALL (S025117, S032957, S026767, ALL-202, ALL-201R, ALL-211, ALL-203, and ALL-212) to 100nmol/L dexamethasone (top) 100nmol/L vincristine (middle), and 10nmol/L daunorubicin (bottom) after 72hours of treatment. (The maximum DMSO concentration reached was 0.2% v/v DMSO.) The PE of AG1024 + U0126 with each drug was determined using paired *t* test analysis. **p ≤ 0.005 indicates moderate synergism and *p ≤ 0.05 indicates synergism.

**Supplementary Figure E1:**
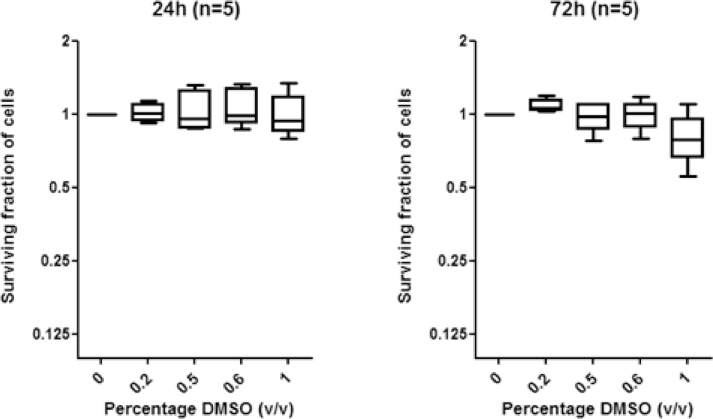
Graphs show minimal effect on survival of cells from 5 representative primary ALL samples following 24h and 72h treatment with doses of DMSO (drug vehicle) reflecting those doses reached within the experimental data (<0.6% DMSO). In contrast, 1% DMSO did cause a cytotoxic effect after 72h.

**Supplementary Figure E2:**
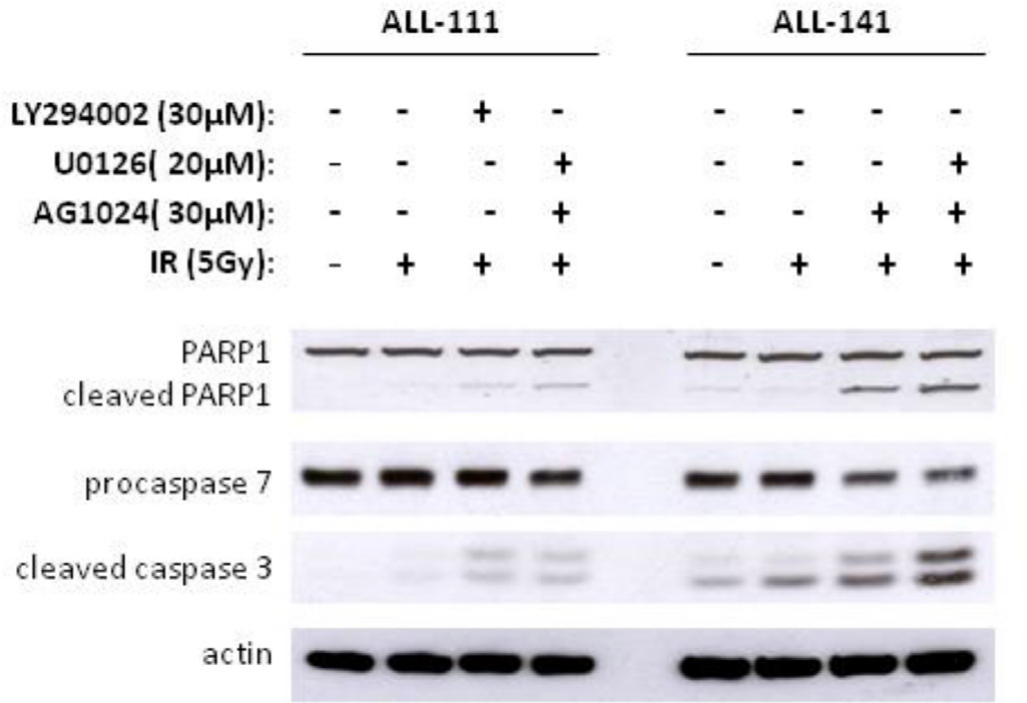
Western blot analysis comparing caspase-dependent apoptosis induced by AG1024+U0126 with the single most potent single inhibitor 8h following treatment in two representative ALL (ALL-111 and ALL-141). In ALL-111, which exhibited equal sensitivity to LY294002 and U0126+AG1024, caspase and PARP cleavage were comparable following 8h treatment. In the completely IR-resistant leukaemia, ALL-141, caspase and PARP1 cleavage was significantly induced by treatment with U0126+AG1024 compared with AG1024 alone, the only single inhibitor with some effect.

**Supplementary Figure E3:**
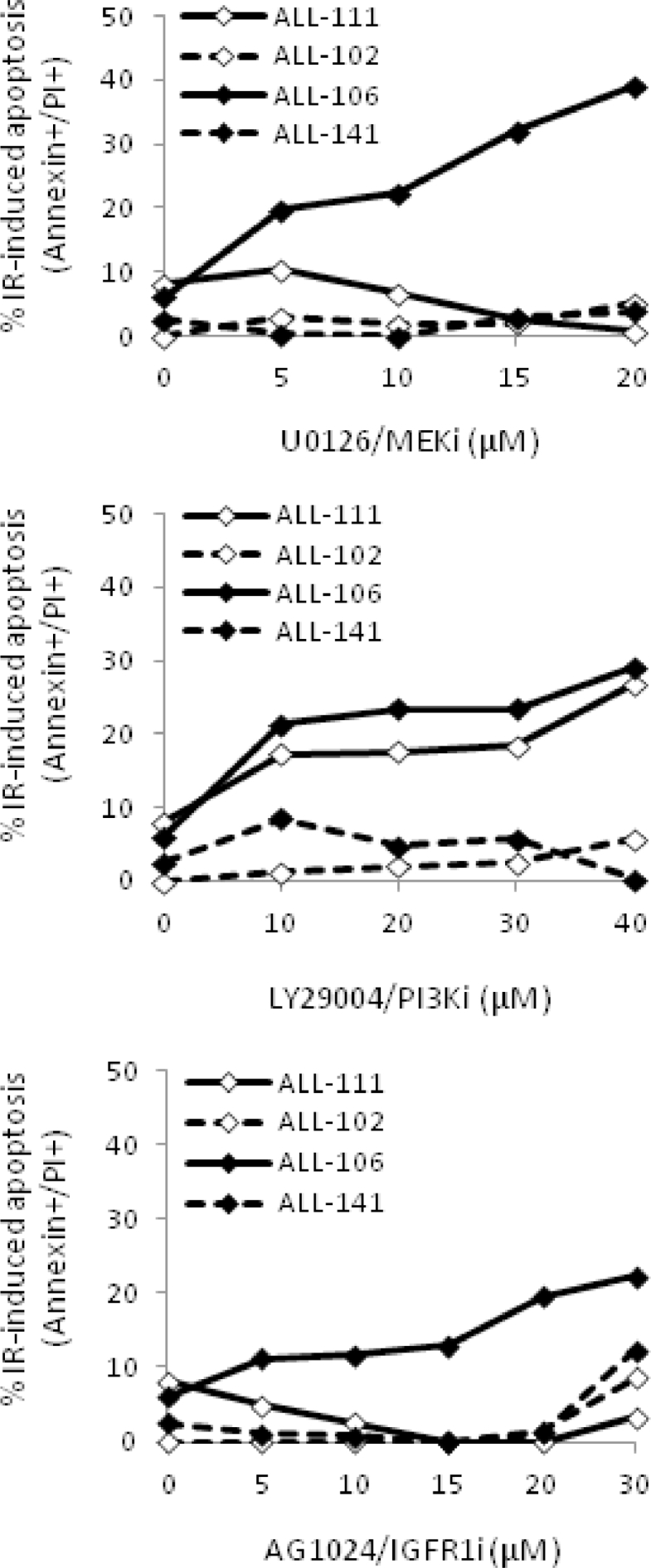
Graphs show dose-curves for U0126 and for AG1024 for four ALLs which were used for Calcusyn analysis to determine the effect of combined U0126+AG1024 treatment.

**Supplementary Figure E4:**
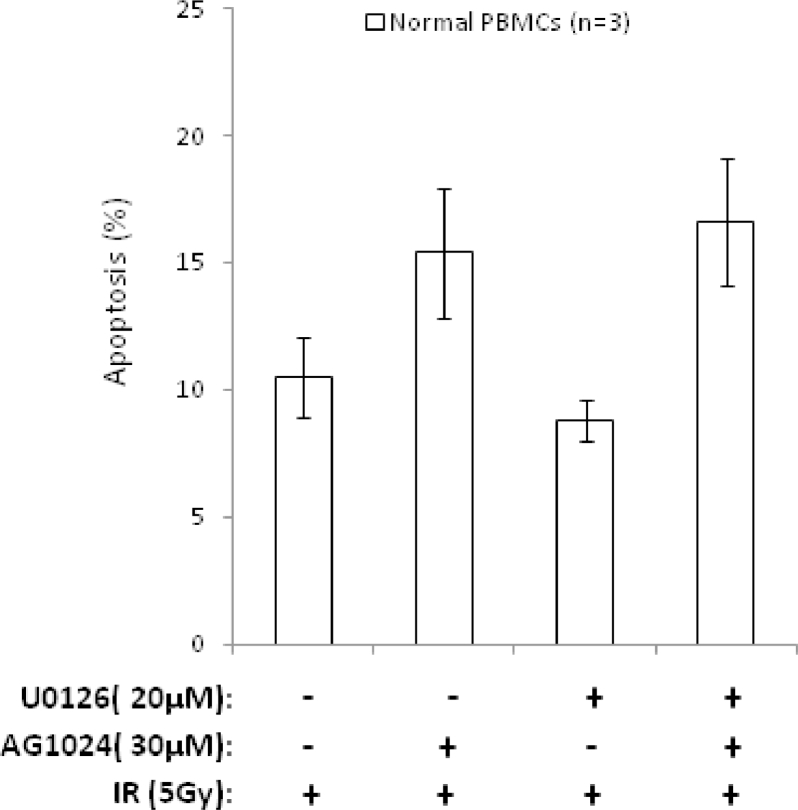
Graph shows absence of a combined effect of AG1024+U1026 on IR-induced apoptosis in peripheral blood mononuclear cells (PBMCs) from three healthy individuals, measured by Annexin V/PI staining and FACS analysis after 72h.

As a consequence of the heterogeneous upregulation of multiple prosurvival signaling pathways underlying defective apoptotic responses in childhood ALL, we reported previously that individual prosurvival pathway inhibitors targeting MEK, IGF1/R, and PI3K induced patient-specific responses to IR-induced DNA damage in vitro 20, 21. We hypothesized that dual combination of prosurvival pathway inhibitors might sensitize a broader range of leukemias and therefore inform of a more applicable therapeutic approach. In this study, we compared dual combinations of the IGF1/R, MEK, and PI3K inhibitors AG1024, U0126, and LY294002, respectively, at the same micromolar doses we reported as single agents in our previous study [21]. We identified that the specific combination of AG1024 (IGF1/R inhibitor) and U0126 (MEK inhibitor) was consistently active in sensitizing four DNA damage-resistant ALLto IR, whereas dual combinations involving thePI3K inhibitor LY294002 induced more variable responses (Figures 1A and B). The combination of AG1024 + U0126 consistently induced the highest level of IR-induced apoptosis, as measured by Annexin and PI staining 24hours after treatment (Figures 1A and B), and this occurred irrespective of the sensitivity of the cells to each single agent [21]. The drug vehicle DMSO alone had minimal effect on the survival of primary ALL cells at the same doses even after 72hours (Supplementary Figure E1, online only, available at www.exphem.org). In two representative samples, western blotting confirmed that AG1024 + U0126 + IR induced caspase- and PARP1-dependent apoptosis to a greater extent than the single most potent inhibitor for each leukaemia. (Supplementary Figure E2, online only, available at www.exphem.org). Using dose– response curves (Supplementary Figure E3, online only, available at www.exphem.org) and Calcusyn software, we were able to determine CI values for ALL-141, ALL-102, and ALL-106, which revealed strong synergism, synergism, and a near additive effect between AG1024 and U0126, respectively (Figure 1A and Supplementary Table E2, online only, available at www.exphem.org). For ALL-111, the dose curves for both U0126 and AG1024 alone were inhibitory. We therefore determined the PE that indicated a strongly synergistic effect of the combined AG1024 + U0126 treatment (Figure 1B and Supplementary Figure E3 and Supplementary Table E2, online only, available at www.exphem.org). Therefore, whereas in ALL-141, ALL-102, and ALL-111, the combined effect of AG1024 + U0126 was synergistic, in ALL-106, the combination of AG1024 + U0126 was not superior to U0126 alone: both single-agent treatments exhibited efficacy, as did the AG1024 + LY294002 combination. Nonetheless, the sensitivity of ALL-106 to AG1024 and U0126 as single agents or in combination shows that a treatment strategy targeting IGF1/R + MEK pathways would sensitize apoptosis-resistant primary ALL cells to IR-induced DNA damage. We observed no synergistic effect of the two agents when combined in normal peripheral blood mononuclear cells in the presence of IR-induced DNA damage (Supplementary Figure E4, online only, available at www.exphem.org).

**Supplementary Table E2:** Combined effect of U0126+AG1024 in ALL treated with IR.

ALL	Combined effect of U0126+AG1024	
ALL-141	CI=0.478	+++ synergism
ALL-102	CI=0.714	++ moderate synergism
ALL-106	CI=1.020	+ nearly additive
ALL-111	PE, p<0.0001	*** very strong synergism

PE, potentiation effect; CI, combination indices.

### IGF1/R + MEK inhibition using AG1024 + U0126 sensitizes childhood ALL to core chemotherapy agents

We next investigated whether this specific combination of IGF1/R + MEK inhibition could sensitize ALL cells to other clinically relevant cytotoxic agents. To do so, we tested whether AG1024 + U0126 would sensitize ALL to the core remission-inducing chemotherapy agents dexamethasone, vincristine, and daunorubicin in vitro. We evaluated the effect of the lower doses 1 and 10 µmol/L AG1024 + U0126 on chemotherapy-induced killing after 72hours in eight primary ALL samples and observed chemosensitization for all three drugs (Figure 1C). In combination with 100nmol/L dexamethasone, synergism was observed with 1 and 10 µmol/L AG1024 + U0126 (PE, paired *t* tests, *p* = 0.0084 and *p* = 0.0166, respectively). AG1024 + U0126 also increased ALL sensitivity to 100nmol/L vincristine, with 1 µmol/L displaying an additive effect and 10 µmol/L displaying synergism (PE, paired *t* test, *p* = 0.019). Finally, AG1024 + U0126 exerted an additive effect with 10nmol/L daunorubicin and, although synergism was observed with 100nmol/L daunorubicin (paired *t* test, *p* = 0.01), the killing induced by daunorubicin alone was already very high and the incremental change was small (not shown). The combination of IGF1/R + MEK inhibition at micromolar doses is therefore able to sensitize ALL cells, including high-risk and relapse samples, to submicromolar doses of chemotherapy agents in vitro.

### Variable basal levels of MEK and IGF1/R activity indicate additional mechanisms underlying the synergistic action of AG1024 + U0126

**Figure2:**
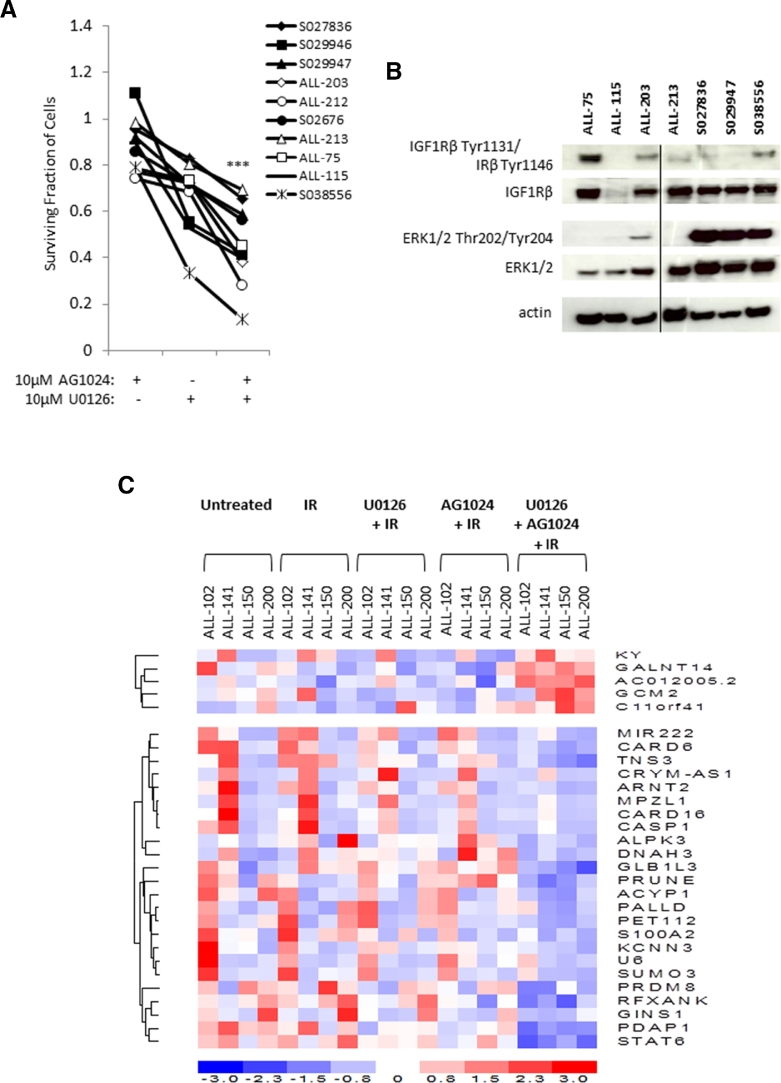
The synergistic effect of combined inhibition of IGF1/R + MEK pathways by AG1024 + U0126 induces a distinct “synergy” gene signature. **(A)** In the absence of cytotoxic agents, primary ALL samples (*n* = 10) display synergistic sensitivity (PE = ***) to treatment with both AG0124 + U0126 after 72hours. (The maximum DMSO concentration reached was 0.2% v/v DMSO.) **(B)** Western blot analysis revealing samples displaying synergism to AG1024 + U0126 and variable basal MEK and IGF1/R pathway activity, as indicated by phosphorylation of IGF1/Rβ + IRβ and ERK1/2, suggesting that additional mechanisms underlie the synergistic effect. **(C)** Heat map showing differential expression of 32 genes (“synergy” signature) induced by 6hours of treatment with AG1024 + U0126 compared with the respective individual inhibitors + IR, IR alone, and untreated cells in ALL-102, ALL-141, ALL-150, and ALL-200. *p ≤ 0.05 indicates synergism; ***p ≤ 0.0005 indicates strong synergism.

**Supplementary Figure E5:**
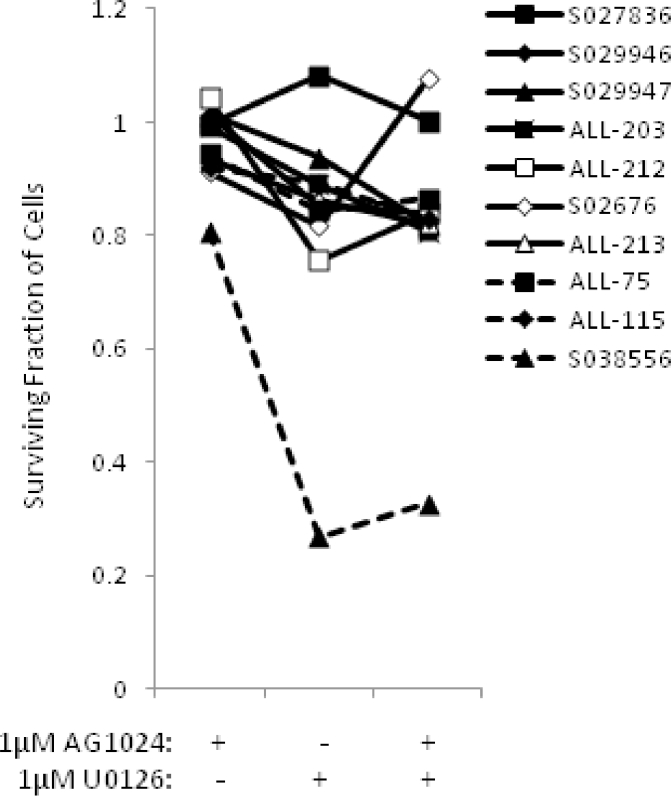
Graph shows no synergism between AG1024 and U0126 at a dose of 1µM following 72h treatment in 10 primary ALL.

We next set about investigating the possible mode of action for the synergistic effect of AG1024 + U0126 in sensitizing childhood ALL cells. To address this, we investigated whether an association existed between the basal levels of the phosphorylated ERK1/2, an indicator of activated MEK pathway, and phosphorylated IGF1/Rβ, an indicator of activated IGF1 and insulin pathways, in 10 primary ALLs that were sensitive to the synergistic effect of 10 µmol/L AG1024 + U0126 after 72hours in vitro compared with each of the single inhibitors in the absence of other cytotoxic agents (PE, paired *t* test, *p* = 0.0001) (Figure 2A). We did not observe a synergistic effect using 1 µmol/L AG1024 + U0126 (Supplementary Figure E5, online only, available at www.exphem.org). These samples (where material was sufficient) revealed highly variable basal levels of phosphorylated ERK1/2 and IGF1/Rβ proteins (Figure 2B), suggesting that the synergistic action of combined AG1024 + U0126 treatment was not wholly dependent on targeting the MEK and IGF1/R pathways.

### Combined AG0124 + U0126 treatment induces a differential “synergy” gene profile

**Supplementary Figure E6:**
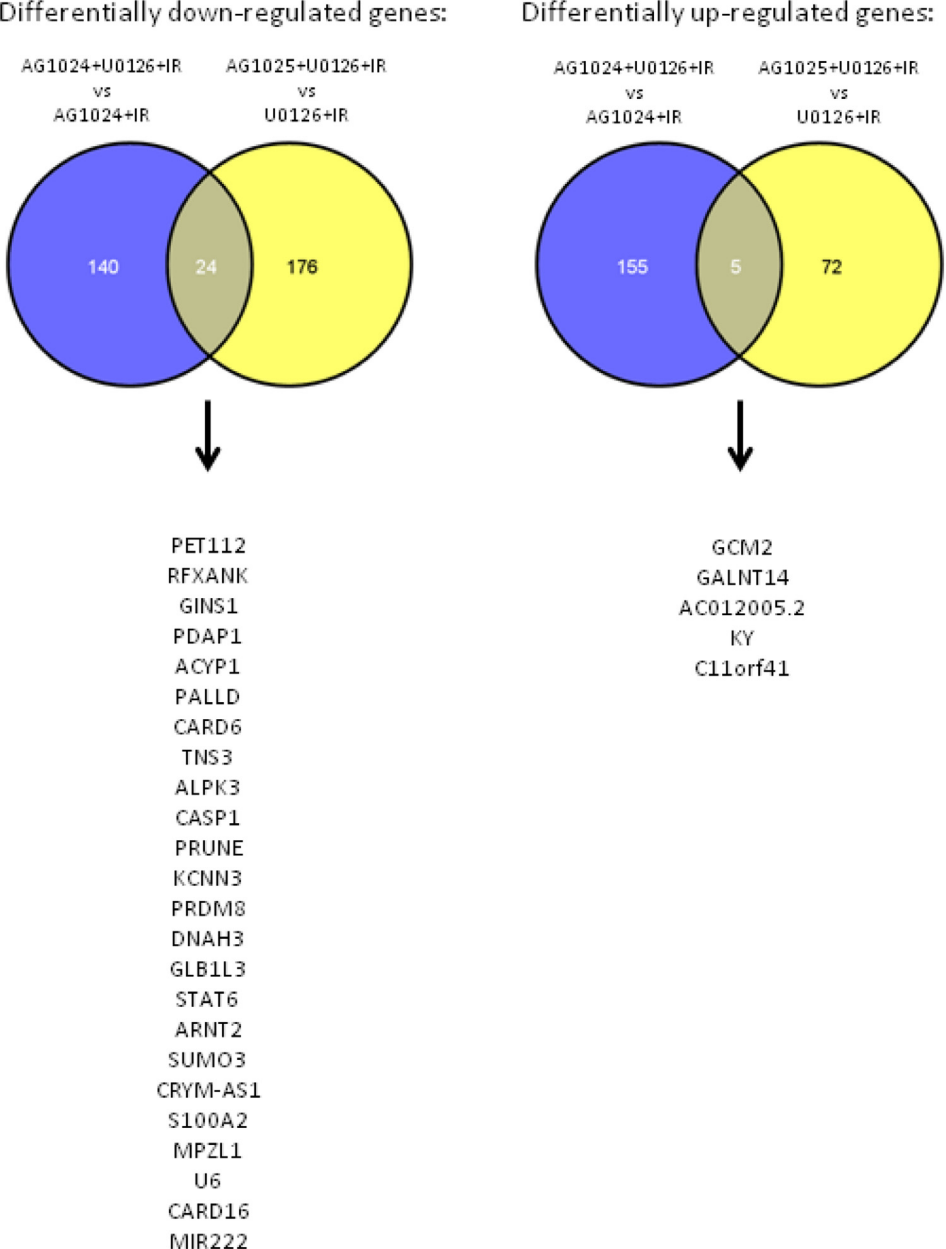
Identification of genes contributing to AG1024+U0126 synergism. Venn analysis identifying ‘synergy’ genes shows 26 genes are differentially down-regulated (left) and 6 genes are differentially upregulated (right) by treatment of ALL cells with AG1024+U0126+IR compared with AG1024+IR and U0126+IR in samples ALL-102, ALL-141, ALL-150 and ALL-200.

Based on the variable basal levels of activated of MEK and IGF1/R pathways, it appears that the synergistic action of AG1024 + U0126 in ALL cells may function in part through additional or off-target activity. To explore possible additional mechanisms of this drug combination, we employed gene expression profiling in four ALLs, ALL-102, ALL-141, ALL-150, and ALL-200, which displayed synergistic responses to AG1024 + U0126. Samples were treated either with IR to induce DNA damage, with each of the single inhibitors + IR, or AG1024 + U0126 + IR for 6hours. To demonstrate that the individual inhibitors were active, we first evaluated the alteration of known target genes by comparing AG1024 + IR with IR alone and U0126 + IR with IR alone. This comparison revealed differential expression of known target genes for each of the individual inhibitors, which, as well as MAPK and G-protein-coupled receptor genes, included *miR222, SP1*, and *DUSP6* 27, 28 for the MEK inhibitor U0126 and *JUN, SPRY, APAF1*, and *BAK1* [29] for the IGF1/R inhibitor AG1024, as well as *TNS3*, previously correlated with IGF1 levels [30]. We subsequently compared gene expression in cells treated with AG1024 + U0126 + IR with cells treated with each inhibitor alone + IR to identify a set of “synergism” genes that were differentially expressed after 6hours of treatment with AG1024 + U0126 (for Venn analysis, see Supplementary Figure E6, online only, available at www.exphem.org). Of the 32 differentially expressed genes, six were consistently differentially upregulated and 26 downregulated by AG1024 + U0126 + IR treatment (Figure 2C). Because this set of genes could contribute to apoptosis resistance in some ALL, we set about investigating candidate synergy genes further.

### Synergy genes *STAT6* and *PDAP1* exhibit codependent expression

**Figure3:**
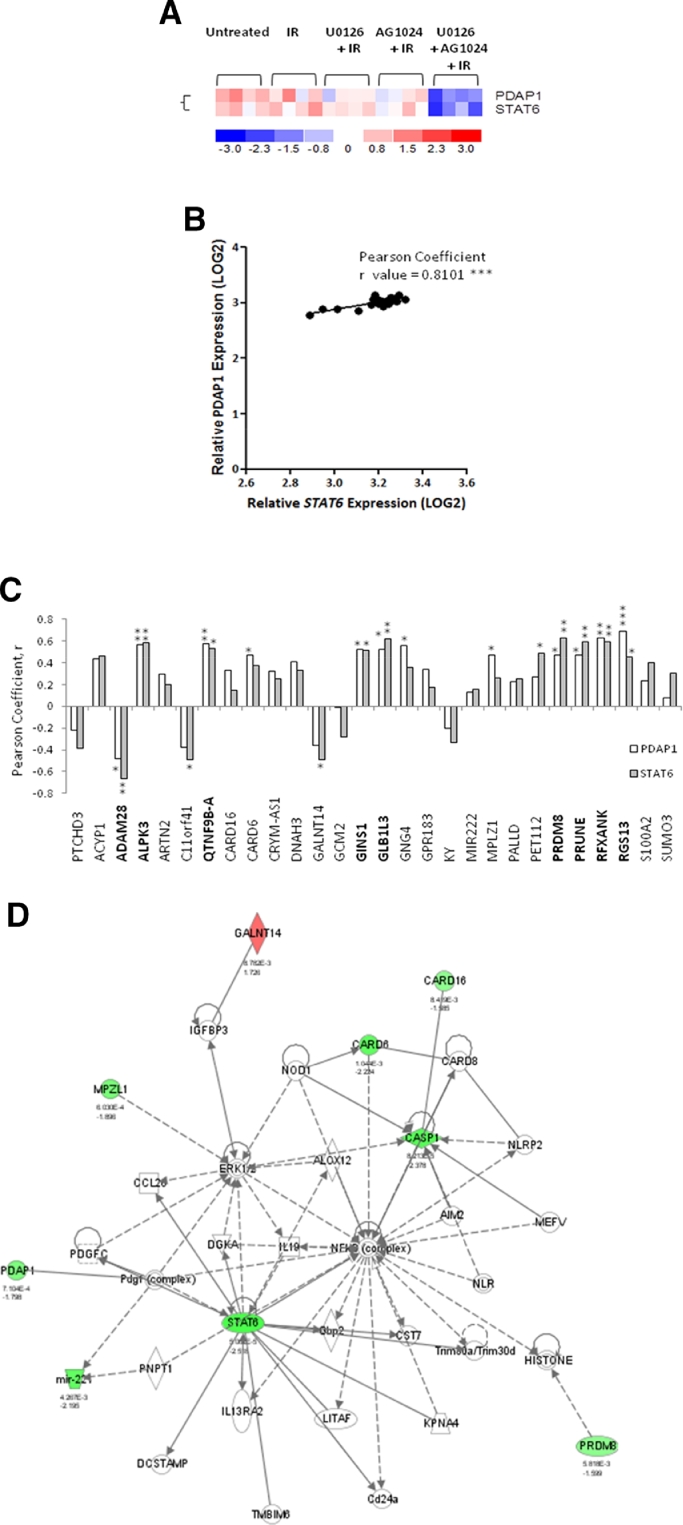
Predicted interactions of “synergy” genes *STAT6* and *PDAP1*. **(A)** Heat map showing that *STAT6* and *PDAP1* are highly differentially downregulated by AG1204 + U0126. **(B)** Pearson correlation coefficient analyses from 20 microarrays indicating that *STAT6* and *PDAP1* expression are highly codependent. **(C)***STAT6* and *PDAP1* display common patterns of expression c-dependency with specific “synergy” genes highlighted in bold text. (Pearson coefficient *r* values: below 0.4 = weak; 0.4–0.6 = modest; above 0.6 = marked codependency). **(D)** Ingenuity network analysis predicting interaction of *PDAP1* and *STAT6* in a STAT6–ERK–NF-κB regulatory network seeded with “synergy” genes that might be involved in apoptosis resistance (green = downregulated; red = upregulated). *p ≤ 0.05; **p ≤ 0.005; ***p ≤ 0.0005.

**Supplementary Figure E7:**
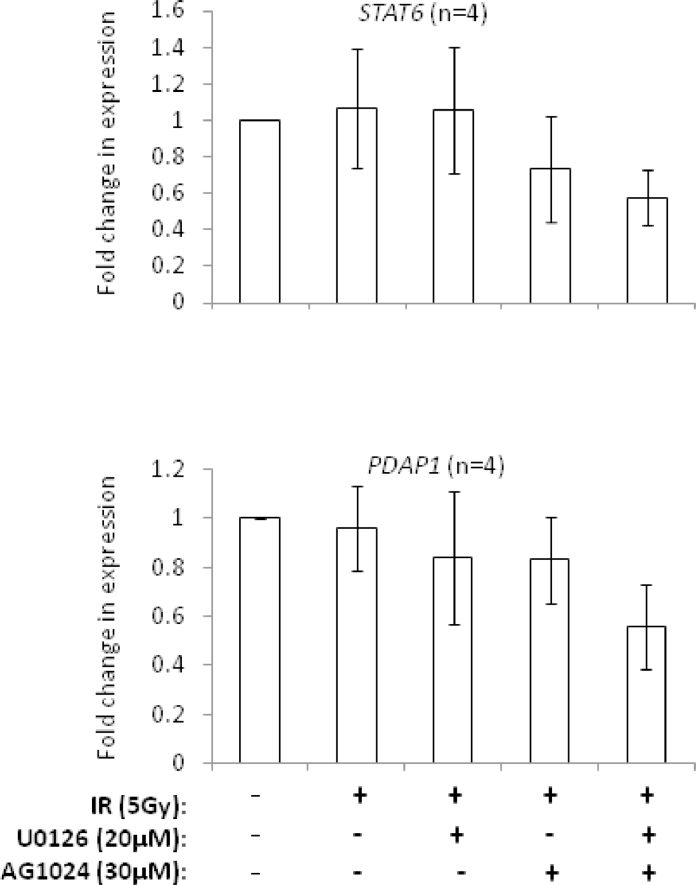
Independent qRT-PCR validation of the most differentially altered ‘synergy’ genes, *STAT6* and *PDAP1*, in ALL employed in the microarray analysis as well as others treated under the same conditions.

Of the set of synergy genes we identified, *STAT6* and *PDAP1* were among the most significantly differentially downregulated (Figure 3A) and were validated by independent quantitative reverse transcription polymerase chain reaction (Supplementary Figure E7, online only, available at www.exphem.org). These genes were of particular interest because of their possible role in high-risk subtypes of childhood ALL. Phosphorylated STAT6 has been reported to be elevated in Ph+ ALL [31] and PDAP1 appears to be involved in PDGF signaling 32, 33, a pathway recently implicated in apoptosis-resistant ALL [21] and Ph-like ALL 8, 9, 10, 11, 12, 18. To provide some insight into how these synergy genes might interact to induce chemosensitization when downregulated, we used logarithmic expression data from 20 arrays used in this study (untreated, IR only, AG1024 + IR, U0126 + IR, and AG1024 + U0126 + IR for each of the four ALL samples) to enable Pearson coefficient correlation analyses. This identified a marked (*r* > 0.6) and highly significant expression codependency between *STAT6* and *PDAP1* (Figure 3B). When we compared a possible relationship between *STAT6* and *PDAP1* and the remaining synergy genes, we observed a striking pattern of linked expression that was common to both *STAT6* and *PDAP1* (Figure 3C).

### Identification of a predicted regulatory network underlying the synergistic activity of AG1024 + U0126 in ALL cells

We next performed network analysis using Ingenuity software to determine whether the synergy genes that we identified were predicted or known to interact in common pathways. This analysis identified a major network associated with cell death and survival, cellular function and maintenance, and hematological system development and function. STAT6 and PDAP1 were associated with this network, of which STAT6 was a major component in addition to ERK and NF-κB. In this STAT6–ERK–NF-κB network, STAT6 and PDAP1 appeared to be closely associated (via *PDGF*), consistent with our codependent expression data (Figure 3D). Other components of this network (*PRDM8* and *MPZL*) had also shown linked expression with both *STAT6* and *PDAP1* using Pearson correlation analysis (see above). This STAT6–ERK–NF-κB network could be an important mechanism underpinning apoptosis resistance in ALL cells and warrants further characterization.

### Knockdown of STAT6 and PDAP1 has an impact on cell cycle and chemosensitization

**Figure4:**
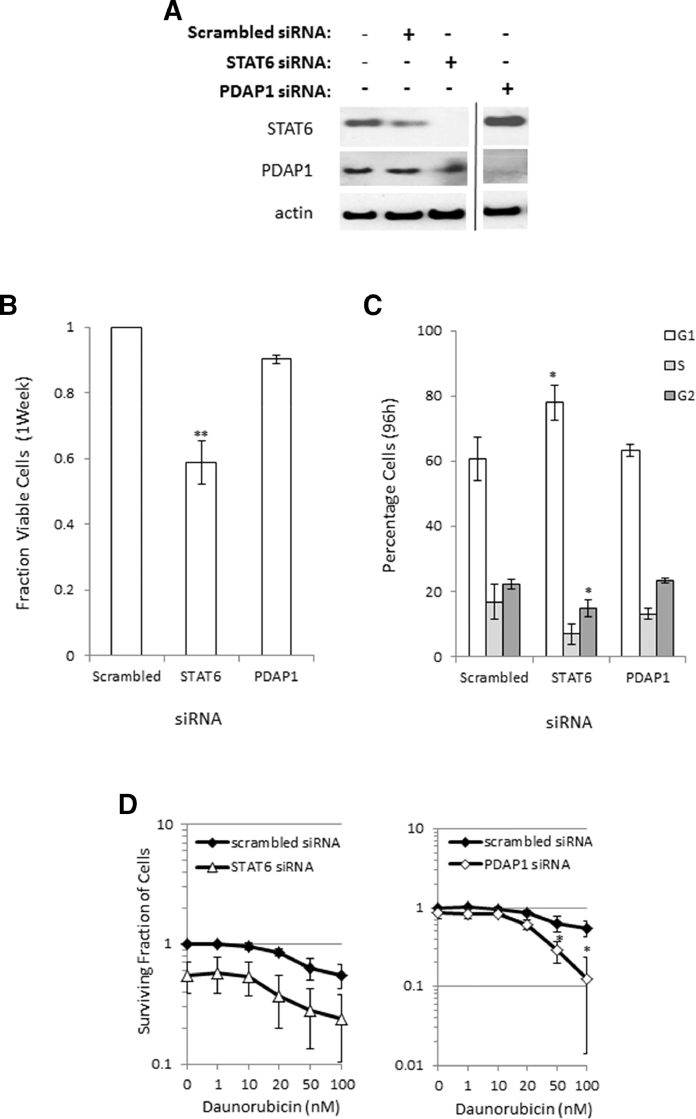
Cellular consequences of siRNA-mediated knockdown of synergy genes. **(A)** Western blot showing siRNA mediated knockdown of STAT6 and PDAP1 in HeLa cells. **(B)** siRNA-mediated knockdown of STAT6 results in a significant loss in cell viability, whereas PDAP1 knockdown has no impact after 1 week. **(C)** siRNA-mediated knockdown of STAT6 causes a 20% increase in cells in the G_1_ phase of the cell cycle, indicating decreased proliferation, whereas PDAP1 knockdown has no impact on the cell cycle in HeLa cells. **(D)** Effect on loss of cell viability by STAT6 knockdown leads to an additive effect with daunorubicin after 72hours of treatment, whereas PDAP1 knockdown is synergistic with daunorubicin at more than one dose in HeLa cells. *p ≤ 0.05; **p ≤ 0.005.

Because our data suggested that STAT6 and PDAP1 might contribute to apoptosis resistance, we hypothesized that loss of these proteins individually might reduce cell viability and/or sensitize cells to chemotherapy. To address the cellular impact of loss of STAT6 and PDAP1, we performed siRNA-mediated knockdown in HeLa cells. Because of the difficulties in obtaining gene knockdown in primary human ALL cells, we used HeLa cells as an alternative since they enable reproducible and effective protein knockdown. Although HeLa cells are of a different tissue origin than ALL, the rationale for choosing this model system to explore the cellular impact of loss of these proteins on chemotherapy sensitization was because they also represent a widely accepted model for studying DNA damage response proteins 34, 35. Figure 4A shows reduction of each of the proteins after 72hours of siRNA treatment. Interestingly, we observed that the loss of PDAP1 appeared to lead to an increase in STAT6 protein expression. This apparent connection at the protein level supports the codependent expression correlations that we observed at the mRNA level for these molecules.

We first investigated the impact of loss of each of the proteins on cell viability and the cell cycle. In untreated cells, STAT6 knockdown by siRNA treatment had the greatest impact on the number of viable cells, whereas PDAP1 loss had no impact on the number of viable cells after 1 week of siRNA treatment (Figure 4B). When we assessed alterations in the cell cycle, we found that, consistent with the reduction in the number of viable cells and with previous findings [36], STAT6 caused a 20% increase in the proportion of cells in the G_1_ phase of the cell cycle, indicating an increased number of arrested cells and a reduction in cycling. In contrast, PDAP1 loss had no impact on the cell cycle, which is consistent with the absence of effect of PDAP1 loss on viable cell number (Figure 4C).

**Supplementary Figure E8:**
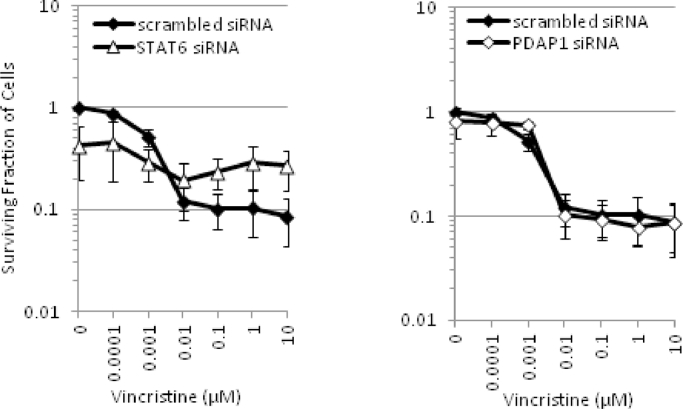
Sensitization of HeLa cells by siRNA silencing of ‘synergy’ candidate genes, *STAT6* and *PDAP1.* Graphs show the decrease in cell proliferation caused by STAT6 knockdown protects HeLa cells from vincristine-induce killing whereas PDAP1 knockdown has no effect compared with scrambled siRNA, following 72h treatment *in vitro*.

We next investigated the ability of loss of each of these proteins to sensitize cells to core chemotherapy agents used to induce remission in ALL patients. When we combined protein knockdown with increasing doses of the anthracycline daunorubicin in vitro, the impact on the total number of viable cells caused by knockdown of STAT6 had an additive effect (Figure 4D), whereas, strikingly, PDAP1 knockdown was synergistic after 72hours (Figure 4D). In contrast, when STAT6 was silenced, the impact on cell cycle arrest (Figure 4D) reduced the sensitivity of HeLa cells to increasing doses of vincristine, the mechanism of which is dependent on cell proliferation. PDAP1 knockdown had no effect at all on vincristine-induced cell killing after 72hours of treatment (Supplementary Figure E8, online only, available at www.exphem.org). The HeLa cells displayed no evidence of sensitivity to dexamethasone with or without knockdown (data not shown). These data support the notion that the inhibition of pathways involving STAT6 and PDAP1 contributes to the potentiating effect of combined AG1024 + U0126 treatment on DNA-damaging agents such as IR and daunorubicin. The inability of loss of the single genes selected for further exploration in this study to sensitize HeLa cells to vincristine or dexamethasone suggests that it is likely that alternative genes or a combination of genes are implicated in sensitization observed in both HeLa and leukemia cells. This is supported by the microarray data. However, with respect to daunorubicin, our data from HeLa cells indicate that STAT6, and in particular PDAP1, appear to function in the cellular response to daunorubicin and warrant further investigation in leukemia cells.

In summary, we have identified a specific combination of prosurvival signaling pathway inhibitors, AG1024 + U0126, which was consistently able to sensitize apoptosis-resistant primary ALL cells to DNA-damaging agents. This dual combination targets a specific set of “synergy” genes that includes *STAT6* and *PDAP1*, which are predicted to function in an STAT6–ERK–NF-κB regulatory network.

## Discussion

Childhood ALL is genetically heterogeneous and deregulation of different prosurvival signaling pathways can contribute to apoptosis resistance. In this study, we sought to determine whether the inhibition of a specific combination of prosurvival pathways could sensitize ALL irrespective of response to single pathway inhibition and if this might inform of a more applicable uniform treatment approach for ALL patients. We have shown that the combined inhibition of the IGF1/R and MEK pathways using AG1024 + U0126 can sensitize ALL cells in a synergistic manner with IR-induced DNA damage and can also potentiate the effects of core chemotherapy agents in vitro. Gene expression profiling revealed a set of synergy genes that included *STAT6* and *PDAP1*, which are predicted to function in a STAT6–ERK–NF-κB regulatory network.

JAK/STAT signaling is implicated in many cancers [36]. STAT6 hyperactivation has been described in a several lymphoid malignancies and, recently, STAT6-activating mutations have been identified in a range of lymphomas with a frequency of up to 40% 36, 37, 38, 39. In ALL, phosphorylated STAT6 levels are elevated in Ph+ disease [31] and, given the similarities, it is interesting to speculate that STAT6 activity might also be upregulated in the high-risk Ph-like ALL subtype. STAT6 signaling has been implicated specifically in treatment resistance and progression in several malignancies, including the response of chronic lymphocytic leukemia cells to B-cell receptor-mediated treatment and radioresistance in inflammatory breast cancer cells 40, 41, 42, 43. There is mounting evidence, therefore, that STAT6 signaling plays a relevant role in the pathophysiology and clinical responses of lymphoid and other malignancies. These data support our conclusions that STAT6 is likely to be important in the cellular response to DNA damage and could contribute to apoptosis resistance in ALL.

Although the function is largely unknown, PDAP1 was originally identified as a PDGF-interacting protein [32]. Indeed, PDAP1 was shown recently to be an effector of PDGR signaling in glioma cells and was associated with proliferation and disease progression, highlighting PDAP1 as a potential therapeutic target [33]. PDGF signaling is a pathway that we recently implicated in apoptosis resistance in childhood ALL [21] and, furthermore, *PDGFRB* translocations are a recurrent feature of high-risk ALL 8, 9, 10, 11, 12, 18. Interestingly, cells with NF1 inactivation and consequential hyperactivation of the RAS–MAPK–ERK signaling pathway also display overexpression of PDGFRs, which contributes to RAS-induced proliferation 44, 45 and supports a role for PDAP1 in a putative STAT6–ERK–NF-κB regulatory network. In our study, we showed that loss of PDAP1 synergized significantly with daunorubicin to induce killing in HeLa cells. We suggest that the role of PDAP1 in the cellular response to DNA-damaging agents should be investigated further in leukemia cells because PDAP1 might represent an interesting novel therapeutic target for chemosensitization.

In summary, through dual IGF1/R and MEK inhibition, we have identified a group of genes that appear to contribute to impaired apoptotic responses to DNA damage and, when targeted, can sensitize ALL cells to chemotherapy agents. In particular, we have demonstrated that STAT6 and PDAP1, via a putative STAT6–ERK–NF-κB network, may represent useful molecular targets for treatment-resistant ALL, particularly in the absence of clinically available IGF1/R inhibitors. It has already been demonstrated that JAK inhibitors such as ruxolitinib or leflunomide, which abrogate JAK3/STAT6 tyrosine phosphorylation, could represent useful treatment approaches for some ALL. A novel therapeutic approach for ALL could also potentially include STAT6 inhibition. STAT6 small-molecule inhibitors are currently under development for the treatment of asthma and could also be evaluated in the context of ALL. Overall, it will be important to delineate STAT6/PDAP1 signaling precisely in ALL and other malignancies to elucidate the role of these molecules in treatment resistance.

## References

[1] Campana D. (2008). Molecular determinants of treatment response in acute lymphoblastic leukemia. Hematology Am Soc Hematol Educ Program.

[2] Campana D. (2009). Minimal residual disease in acute lymphoblastic leukemia. Semin Hematol.

[3] Mitchell C, Payne J, Wade R, Vora A, Kinsey S, Richards S, Eden T (2009). The impact of risk stratification by early bone-marrow response in childhood lymphoblastic leukaemia: results from the United Kingdom Medical Research Council trial ALL97 and ALL97/99. Br J Haematol.

[4] Schrappe M, Hunger SP, Pui CH (2012). Outcomes after induction failure in childhood acute lymphoblastic leukemia. N Engl J Med.

[5] Roy A, Cargill A, Love S (2005). Outcome after first relapse in childhood acute lymphoblastic leukaemia: lessons from the United Kingdom R2 trial. Br J Haematol.

[6] Lawson S, Harrison G, Richards S (2000). The UK experience in treating relapsed childhood acute lymphoblastic leukaemia: a report on the medical research council UKALLR1 study. Br J Haem.

[7] Einsiedal HG, von Stackelberg A, Hartmann R (2008). Long-term outcome in children with relapsed ALL by risk-stratified salvage therapy: results of trial acute lymphoblastic leukaemia: relapse study of the Berlin-Frankfurt-Munster Group 87. J Clin Oncol.

[8] Tasian SK, Hunger SP (2017). Genomic charactertization of paediatric acute lymphoblastic leukaemia: an opportunity for precision medicine therapeutics. Br J Haematol.

[9] Den Boer ML, van Slegtenhorst M, De Menezes RX (2009). A subtype of childhood acute lymphoblastic leukaemia with poor treatment outcome: a genome-wide classification study. Lancet Oncol.

[10] Roberts KG, Morin RD, Zhang J (2012). Genetic alterations activating kinase and cytokine receptor signaling in high-risk acute lymphoblastic leukemia. Cancer Cell.

[11] Schwab CJ, Chilton L, Morrison H (2013). Genes commonly deleted in childhood B-cell precursor acute lymphoblastic leukemia: association with cytogenetics and clinical features. Haematologica.

[12] Harrison C. (2013). Targeting signaling pathways in acute lymphoblastic leukemia: new insights. Hematology Am Soc Hematol Educ Program.

[13] Mullighan CG, Su X, Zhang J CB (2009). Deletion of IKZF1 and prognosis in acute lymphoblastic leukemia. N Engl J Med.

[14] Holmfeldt L, Wei L, Diaz-Flores E (2013). The genomic landscape of hypodiploid acute lymphoblastic leukemia. Nat Genet.

[15] Case M, Matheson E, Minto L (2008). Mutation of genes affecting the RAS pathway is common in childhood acute lymphoblastic leukemia. Cancer Res.

[16] Maude SL, Tasian SK, Vincent T (2012). Targeting JAK1/2 and mTOR in murine xenograft models of Ph-like acute lymphoblastic leukemia. Blood.

[17] Tasian SK, Doral MY, Borowitz MJ (2012). Aberrant STAT5 and PI3K/mTOR pathway signaling occurs in human CRLF2-rearranged B-precursor acute lymphoblastic leukemia. Blood.

[18] Weston BW, Hayden MA, Roberts KG (2013). Tyrosine kinase inhibitor therapy induces remission in a patient with refractory EBF1-PDGFRB-positive acute lymphoblastic leukemia. J Clin Oncol.

[19] Eyre T, Schwab CJ, Kinstrie R (2012). Episomal amplification of NUP214-ABL1 fusion gene in B-cell acute lymphoblastic leukemia. Blood.

[20] Weston VJ, Austen B, Wei W (2004). Apoptotic resistance to ionizing radiation in pediatric B-precursor acute lymphoblastic leukemia frequently involves increased NF-kappaB survival pathway signaling. Blood.

[21] Marston E, Weston V, Jesson J (2009). Stratification of pediatric ALL by in vitro cellular responses to DNA double-strand breaks provides insight into the molecular mechanisms underlying clinical response. Blood.

[22] Bolstad BM, Irizarry RA, Astrand M, Speed TP (2003). A comparison of normalization methods for high density oligonucleotide array data based on variance and bias. Bioinformatics.

[23] Irizarry RA, Bolstad BM, Collin F, Cope LM, Hobbs B, Speed TP (2003). Summaries of Affymetrix GeneChip probe level data. Nucleic Acids Res.

[24] Emig D, Salomonis N, Baumbach J, Lengauer T, Conklin BR, Albrecht M (2010). AltAnalyze and DomainGraph: analyzing and visualizing exon expression data. Nucleic Acids Res.

[25] Smyth GK (2004). Linear models and empirical bayes methods for assessing differential expression in microarray experiments. Stat Appl Genet Mol Biol.

[26] Volpe G, Walton DS, Del Pozzo W (2013). C/EBPα and MYB regulate FLT3 expression in AML. Leukemia.

[27] Couts KL, Anderson EM, Gross MM, Sullivan K, Ahn NG (2013). Oncogenic B-Raf signaling in melanoma cells controls a network of microRNAs with combinatorial functions. Oncogene.

[28] Kennedy RA, Kemp TJ, Sugden PH, Clerk A (2006). Using U0126 to dissect the role of the extracellular signal-regulated kinase 1/2 (ERK1/2) cascade in the regulation of gene expression by endothelin-1 in cardiac myocytes. J Mol Cell Cardiol.

[29] Dupont J, Dunn SE, Barrett JC, LeRoith D (2003). Microarray analysis and identification of novel molecules involved in insulin-like growth factor-1 receptor signaling and gene expression. Recent Prog Horm Res.

[30] Teumer A, Qi Q, Nethander M (2016). Genomewise meta-analysis identified loci associated with IGF-1 and IGFBP-3 levels with impact on age-related traits. Aging Cell.

[31] Demehri S, O'Hare T, Eide CA (2010). The function of the pleckstrin homology domain in BCR–ABL-mediated leukemogenesis. Leukemia.

[32] Fischer WH, Schubert D (1996). Characterization of a novel platelet-derived growth factor-associated protein. J Neurochem.

[33] Sharma V, Singh A, Srivastava S (2016). Increased expression of platelet-derived growth factor associated protein-1 is associated with PDGF-B mediated glioma progression. Int J Biochem Cell Biol.

[34] Townsend K, Mason H, Blackford AN (2009). Mediator of DNA damage checkpoint 1 (MDC1) regulates mitotic progression. J Biol Chem.

[35] Stewart GS, Maser RS, Stankovic T (1999). The DNA double-strand break repair gene hMRE11 is mutated in individuals with an ataxia-telangiectasia-like disorder. Cell.

[36] Bruns HA, Kaplan MH (2006). The role of constitutively active Stat6 in leukaemia in and lymphoma. Crit Rev Oncol Hematol.

[37] Yildiz M, Li H, Bernard D (2015). Activating STAT6 mutations in follicular lymphoma. Blood.

[38] Ritz O, Guiter C, Castellano F (2009). Recurrent mutations of the STAT6 DNA binding domain in primary mediastinal B-cell lymphoma. Blood.

[39] Spina V, Bruscaggin A, Cuccaro A, et al. Circulating tumor DNA reveals genetics, clonal evolution and residual disease in classical Hodgkin lymphoma. Blood. 2018 [Epub ahead of print]. 10.1182/blood-2017-11-812073 29449275

[40] Aguilar-Hernandez MM, Blunt MD, Dobson R (2016). IL-4 enhances expression and function of surface IgM in CLL cells. Blood.

[41] Rahal OM, Wolfe AR, Mandal PK (2017). Blocking interleukin (IL)4- and IL13-mediated phosphorylation of STAT6 (Tyr641) decreases M2 polarization of macrophages and protects againstmacrophage-mediated radio-resistance of inflammatory breast cancer. Int J Radiat Oncol Biol Phys.

[42] Lu G, Shi W, Zheng H (2018). Inhibition of STAT6/Anoctamin-1 activation suppresses proliferation and invasion of gastric cancer cells. Cancer Biother Radiopharm.

[43] Nappo G, Handle F, Santer FR (2017). The immunosuppressive cytokine interleukin-4 increases the clonogenic potential of prostate stem-like cells by activation of STAT6 signalling. Oncogenesis.

[44] Badache A, De Vries GH (1998). Neurofibrosarcoma-derived Schwann cells overexpress platelet-derived growth factor (PDGF) receptors and are induced to proliferate by PDGF BB. J Cell Physiol.

[45] Holtkamp N, Mautner VF, Friedrich RE (2004). Differentially expressed genes in neurofibromatosis 1-associated neurofibromas and malignant peripheral nerve sheath tumors. Acta Neuropathol.

